# Case Report: Fournier's gangrene occurring in the setting of intestinal obstruction complicated by suspected intra-abdominal infection, successfully managed with repeated surgical debridement

**DOI:** 10.3389/fsurg.2026.1890179

**Published:** 2026-09-04

**Authors:** Tianyu Wu, Na Hu, Yixiang Ma

**Affiliations:** 1 Department of Urology, Peking University First Hospital, Beijing, China; 2 Department of Geriatrics, Beijing Xicheng District Ping’an Hospital, Beijing, China; 3 Department of Emergency, Peking University First Hospital, Beijing, China

**Keywords:** case report, emergency medicine, fournier's gangrene, necrotizing fasciitis, surgical debridement

## Abstract

**Background:**

Fournier's gangrene (FG) is an uncommon but rapidly progressive necrotizing soft-tissue infection involving the perineum, external genitalia, and perianal region. As a time-critical urological and emergency condition, it requires prompt recognition, broad-spectrum antimicrobial therapy, metabolic stabilization, and urgent surgical source control.

**Case summary:**

We report a 47-year-old man who developed fever, marked scrotal swelling, skin breakdown, and purulent discharge after an episode of intestinal obstruction complicated by possible intra-abdominal infection. FG was diagnosed on the basis of the clinical presentation, microbiological findings, and abdominopelvic computed tomography showing extensive gas-forming infection extending from the scrotum to the pelvis, abdominal wall, and retroperitoneal structures. Cultures from blood and wound exudate grew Klebsiella pneumoniae. The patient received integrated treatment comprising broad-spectrum antimicrobial therapy, glycaemic control, and repeated surgical debridement. After more than two months of treatment, his symptoms markedly improved, with satisfactory wound healing.

**Conclusion:**

This case describes FG arising after intestinal obstruction complicated by suspected intra-abdominal infection, with extensive perineal and scrotal wounds already present at admission and wide anatomical spread of infection confirmed intraoperatively. The patient recovered gradually after repeated radical surgical debridement and broad-spectrum antimicrobial therapy. The diagnostic course and surgical strategy in this case may offer practical insight into the management of extensive FG.

## 1 Introduction

Fournier's gangrene (FG) is a necrotizing fasciitis of the perineal, genital, and perianal regions. The condition was named after the French venereologist Jean-Alfred Fournier, who described fulminant gangrene of the male genitalia in 1883 (1). FG predominantly affects middle-aged and older men, with a reported male-to-female ratio of approximately 10:1 and a peak incidence between 40 and 59 years of age (2). Despite advances in critical care and antimicrobial therapy, FG remains a high-mortality surgical emergency, with contemporary population-level analyses reporting an overall mortality of approximately 19.8% (3). The clinical spectrum ranges from local erythema, swelling, and pain to skin necrosis, wound breakdown, sepsis, and septic shock. Necrotizing infection can spread rapidly along fascial planes into the abdominal wall, back, and retroperitoneum, producing life-threatening systemic illness (4). Established precipitating factors include diabetes mellitus, immunodeficiency, alcohol misuse, and urogenital or anorectal disease. Diabetes mellitus is particularly important and is present in approximately 30%–40% of patients with FG (5).

Diagnosis is primarily clinical and is supported by laboratory and imaging findings. Early manifestations can be nonspecific, and prodromal scrotal swelling or perineal pain may precede overt skin necrosis (6, 7). Computed tomography (CT) is the preferred imaging modality when the diagnosis or extent of disease is uncertain. Characteristic findings include subcutaneous or fascial gas, fascial thickening, and soft-tissue edema; reported diagnostic sensitivity and specificity are approximately 85%–95% in appropriate clinical contexts (8). Management requires resuscitation, broad-spectrum antimicrobial therapy, metabolic optimization, and prompt, radical surgical debridement. Among these interventions, timely operative source control is the decisive determinant of outcome (9).

This case report describes a patient who developed FG possibly secondary to intestinal obstruction and suspected intra-abdominal infection, a precipitating factor that, to our knowledge, has not been previously reported. Although there is no direct clinical evidence confirming that FG was directly caused by intestinal obstruction and intra-abdominal infection, this case may provide new insights into research on the precipitating factors of FG. In addition, conventional severity prediction scores did not classify this patient as high risk; however, intraoperative exploration revealed extensive anatomical spread of infection. We therefore suggest that more multidimensional and standardized risk prediction tools are needed to guide the diagnosis and treatment of patients with FG. Meanwhile, the multiple surgical procedures and prolonged clinical course in this case may provide useful experience and considerations for the clinical management of patients with extensive FG.

## 2 Case presentation

### 2.1 Patient history

A 47-year-old man presented to the emergency department with a 5-day history of fever, progressive scrotal swelling, skin breakdown, and purulent discharge. His maximum recorded temperature was 38.0 °C. Before onset of the perineoscrotal symptoms, he had been treated at another hospital for several days of abdominal pain and distension, vomiting, and cessation of flatus and defecation. Initial blood tests showed inflammatory changes and non-contrast abdominopelvic CT report at the referring hospital showed multiple fluid-filled and dilated small-bowel loops with air-fluid levels, with the transition point located at the terminal ileum; abdominopelvic fluid accumulation and edema or exudative changes in the abdominopelvic fat planes, supporting mechanical small-bowel obstruction complicated by intra-abdominal infection. The precise cause remained unclear because he had no history of abdominal surgery and CT showed no intraluminal foreign body, obvious bowel-wall lesion, or extrinsic compressive mass. No imaging evidence of bowel ischemia, necrosis, perforation, abscess, or fistula was documented, and no intra-abdominal fluid or tissue was cultured. His abdominal symptoms improved after conservative management, including fasting, nasogastric decompression, and enema therapy. The patient reported that scrotal swelling and skin breakdown were absent during the initial obstructive episode. On the day of discharge, he developed fever and scrotal swelling with skin breakdown. Local gas-forming bacterial infection was suspected, and intravenous antimicrobial therapy was initiated at a local hospital; however, the response was inadequate, and he was transferred to our institution. The patient had a 5-year history of type 2 diabetes mellitus treated with oral metformin and acarbose. He did not monitor blood glucose regularly, and his baseline glycemic control was unknown. He had no notable personal or family history.

### 2.2 Physical examination

**Figure 1:**
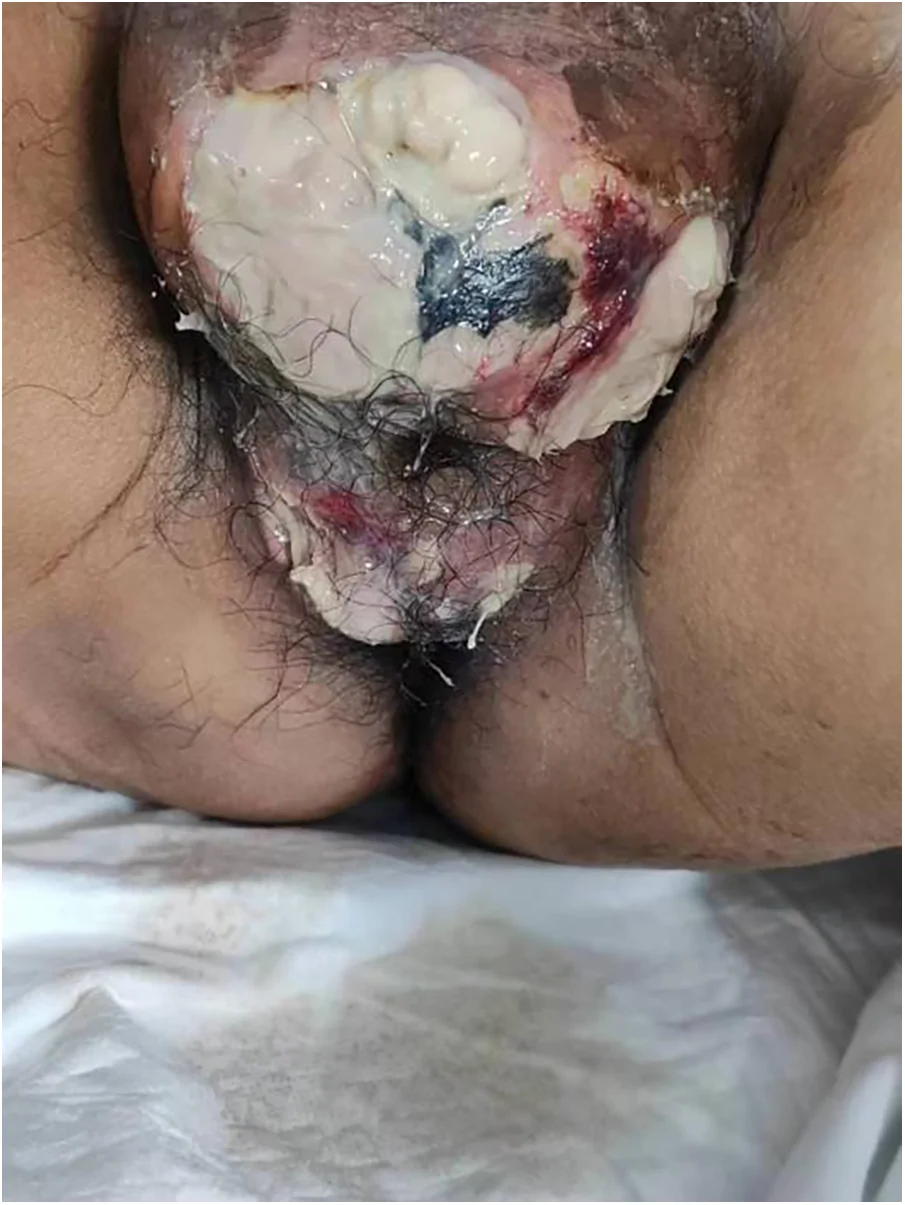
Physical examination on admission showed marked scrotal swelling with skin breakdown and purulent discharge.

On arrival, the patient was alert and able to answer questions. Vital signs were as follows: body temperature, 37.9 °C; heart rate, 113 beats/min; respiratory rate, 22 breaths/min; and blood pressure, 128/91 mmHg. Abdominal examination revealed no tenderness, rebound tenderness, or guarding. Genital examination showed marked scrotal enlargement with multiple areas of skin breakdown, purulent discharge, and pronounced local tenderness (Figure 1). No other major abnormalities were identified.

### 2.3 Laboratory and microbiological findings

Initial laboratory tests showed anemia and systemic inflammation: hemoglobin, 99 g/L (reference, 130–175 g/L); leukocyte count, 16.9 × 10^9/L (reference, 3.50–9.50 × 10^9/L); neutrophils, 92.5% (reference, 50%–70%); C-reactive protein, 123 mg/L (reference, <6.0 mg/L); and procalcitonin, 0.38 ng/mL (reference, <0.15 ng/mL). Serum albumin was 29 g/L (reference, 35–55 g/L), glucose was 12.5 mmol/L (reference, 3.9–6.1 mmol/L), sodium was 133 mmol/L (reference, 135–145 mmol/L), potassium was 3.3 mmol/L (reference, 3.5–5.5 mmol/L), and creatinine was 40 μmol/L. Arterial blood-gas analysis at admission showed pH was 7.30, PaCO2 was 32 mmHg, HCO3− was 18.0 mmol/L, base excess was −6.2 mmol/L, and lactate was 2.1 mmol/L. Coagulation testing and urinalysis showed no significant abnormalities.

Blood, urine, and wound exudate samples were collected for culture and antimicrobial susceptibility testing. Blood and wound cultures grew Klebsiella pneumoniae, which was susceptible to meropenem, ceftazidime, and tigecycline but resistant to levofloxacin. Urine culture was negative.

### 2.4 Imaging findings

**Figure 2:**
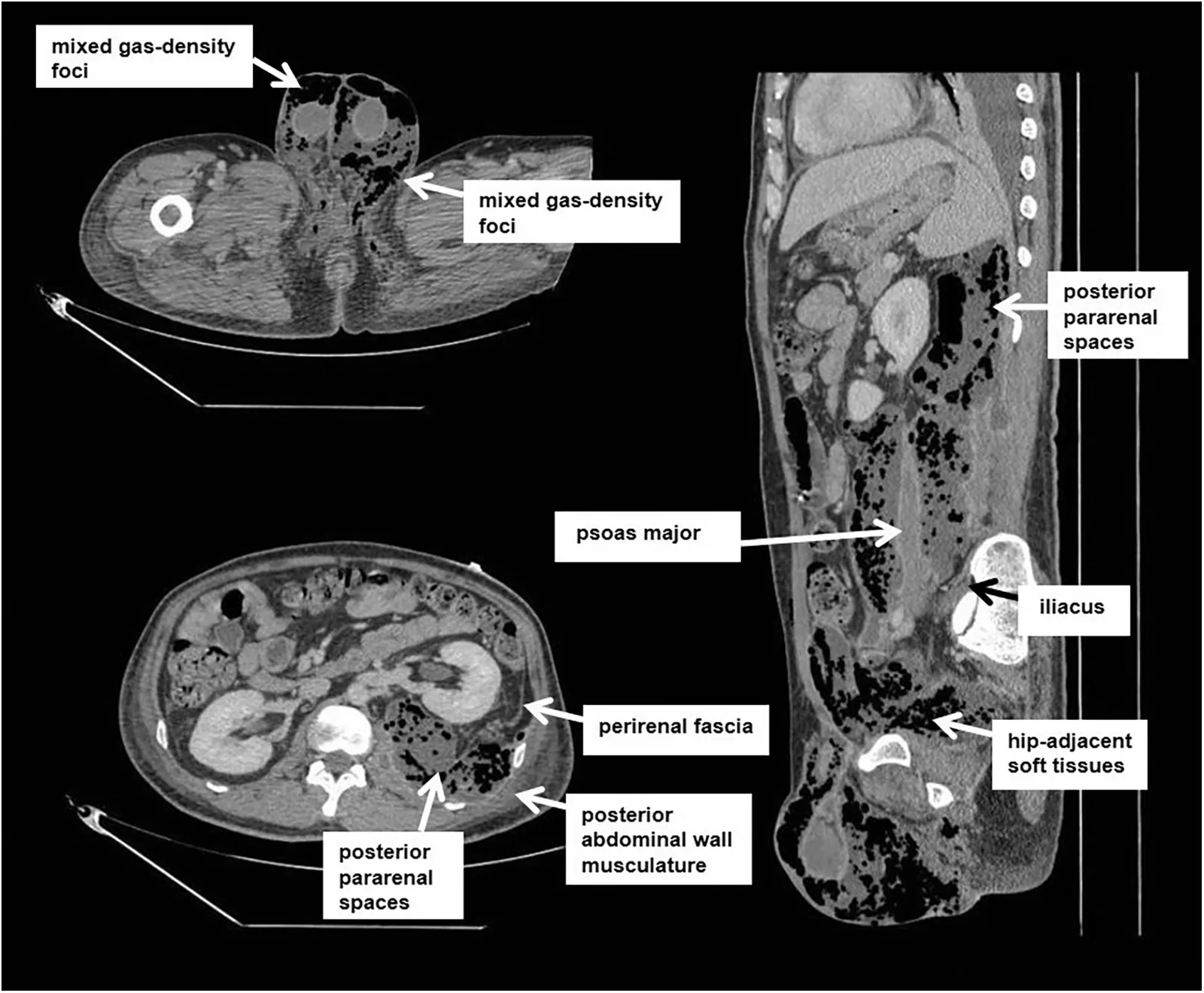
Contrast-enhanced abdominopelvic CT showed bilateral scrotal swelling with mixed gas-density lesions extending superiorly to the pelvis and abdominal wall, with involvement of the left psoas and iliacus muscles, left perirenal fascia, and perirenal spaces, indicating extensive disease.

Scrotal ultrasonography showed abnormal echogenicity in the periscrotal soft tissues, raising concern for infection with gas formation. Non-contrast abdominopelvic CT revealed bilateral scrotal enlargement with multiple mixed gas-density foci extending superiorly to the pelvis and abdominal wall. A mixed-density mass-like lesion was present in the left mid-abdomen, with involvement of the left psoas and iliacus muscles. The process extended through the left perirenal fascia into the left perirenal and posterior pararenal spaces and involved the left posterior abdominal wall, transversus abdominis, internal oblique, and external oblique muscles. Contrast-enhanced CT further demonstrated perianal soft-tissue irregularity with adjacent gas, bilateral scrotal swelling with intralesional gas extending into the pelvis and abdominal wall, involvement of the left psoas and iliacus muscles, extension to the left perirenal and posterior pararenal spaces, and involvement of the left hip-adjacent soft tissues (Figure 2). These findings supported extensive emphysematous necrotizing infection consistent with FG.

### 2.5 Treatment and clinical course

The initial clinical diagnosis was FG. Possible precipitating factors included the patient's history of diabetes mellitus. Based on the examination findings obtained at the referring hospital and the microbiological culture results after admission, intestinal obstruction complicated by suspected intra-abdominal infection was also considered a possible precipitating factor. The patient was admitted, placed nil per os, and treated with intravenous fluid resuscitation, local wound disinfection, strict glycemic control, and empirical intravenous meropenem plus vancomycin.

**Figure 3:**
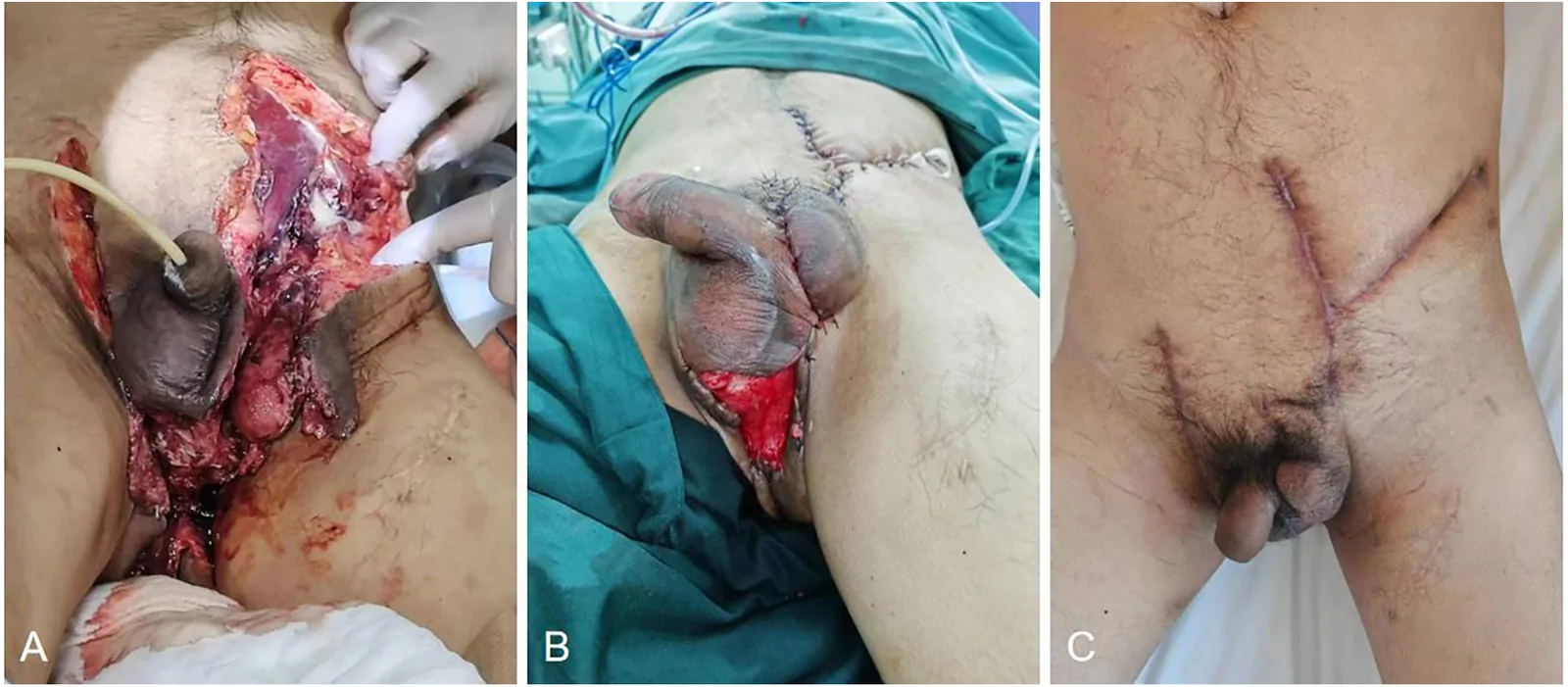
Wound management and healing during treatment. **(A)** Initial emergency debridement after admission, with exploration of the involved compartments and removal of necrotic tissue and purulent material. **(B)** During the third abdominal and perineal wound exploration, wound perfusion and infection control were reassessed, selected wounds were partially closed, and scrotal and lumbar wounds were left for staged closure. **(C)** After serial surgery and wound care, local wound healing was satisfactory at discharge.

Within 12 h of admission, emergency exploration and debridement of the perineal and abdominal wounds were performed under general anesthesia. Intraoperatively, two necrotic perineoscrotal soft-tissue defects were identified, with extensive necrotic fascia and purulent discharge. The two wounds communicated through a subscrotal cavity. Exploration showed inferior extension toward the perianal region and superior extension to the left testis, bilateral inguinal regions, and lower abdominal wall. Purulent material was identified beneath the superficial abdominal fascia and deep to the rectus abdominis. The wound tracked medially toward the umbilical midline and laterally to the left iliolumbar region. In the left iliolumbar area, infection extended along the iliac bone toward the posterior pelvis. Portions of the left posterior abdominal wall musculature, including the transversus abdominis and oblique muscles, were nonviable. Because CT had demonstrated involvement of the psoas and iliacus muscles, perirenal fascia, and retroperitoneal spaces, the surgically accessible retroperitoneal compartments were explored and opened, purulent material and devitalized tissue were removed. Clearly necrotic tissue and purulent material were removed while viable tissue was preserved where possible. After hemostasis, the wounds were sequentially irrigated with hydrogen peroxide solution, normal saline, and povidone-iodine solution, followed by repeated saline irrigation. The wounds were packed with gauze and covered (Figure 3A).

After the initial emergency debridement, the patient remained afebrile and hemodynamically stable, and the leukocyte count, neutrophil percentage, C-reactive protein, and procalcitonin gradually decreased. The wounds were managed openly with daily assessment, cleansing and irrigation, povidone-iodine-based local antisepsis, gauze dressing changes, and reassessment of tissue viability by the burn and plastic surgery team. The exposed wound surfaces showed no rapid extension of necrosis or uncontrolled infection. Because the extensive anatomical involvement identified on CT and at the first operation made repeated staged management likely, the patient subsequently underwent three planned abdominal and perineal wound explorations with extended debridement, partial closure, and drain placement on postoperative days 20, 30, and 40. During these procedures, areas of fresh wound bed and granulation tissue were observed, but deeper regions still contained residual necrotic tissue and localized purulent material. Further debridement was performed, obvious necrosis and purulence were removed, viable tissue was preserved, granulation tissue was refreshed to the fibrous layer, hemostasis was secured, and the wounds were irrigated. Selected wounds were partially closed, and drains were placed after intraoperative reassessment (Figure 3B). During the first extended debridement, drainage strips were placed beneath the partially closed lower abdominal wound and the perineal wound. The drainage was scant and consisted of light yellow serous fluid; therefore, the drains were removed 3 days after the first extended debridement. Following debridement of the deeper tissues in the lower back, where evidence of infection was identified intraoperatively, a rubber drainage tube was placed. During the subsequent second extended debridement, the position of the drainage tube was adjusted, and both the characteristics and volume of the drainage were closely monitored postoperatively. Because the drainage remained consistently bloody after the patient returned to the ward and two consecutive drainage fluid cultures were negative, no irrigation of the drainage tube was performed. Considering that prolonged indwelling drainage might adversely affect wound healing, the drainage tube in the lower back incision was removed 4 days after the second extended debridement. After surgery, the burn and plastic surgery team managed the wounds with an open approach, including wound assessment, cleaning, normal saline irrigation, local antimicrobial treatment with povidone-iodine preparations, wet-to-dry gauze dressing changes, and evaluation of tissue viability two to three times daily.

On postoperative day 60 after the initial operation, the patient underwent final wound revision, closure, and local flap transfer. The left lumbar and dorsal wound bed was fresh with red granulation tissue and no evident necrosis or purulent discharge. The perineoscrotal wound was clean without visible necrosis. Granulation tissue was refreshed until punctate bleeding was observed, wound margins were revised, and drainage tubes were placed after hemostasis and irrigation. Because direct closure of the perineoscrotal wound created excessive tension, a local Z-plasty flap was designed, rotated to cover the defect, and sutured after hemostasis; a drainage strip was left in place.

Throughout surgical treatment, antimicrobial therapy was adjusted according to culture and susceptibility results. Empirical meropenem plus vancomycin was initiated on admission. After blood and wound-exudate cultures grew K. pneumoniae susceptible to meropenem and ceftazidime, vancomycin was discontinued and meropenem was continued at 1 g every 8 h. Necrotic tissue and purulent material obtained during the initial emergency operation also grew K. pneumoniae with a similar susceptibility profile. At the first staged extended debridement on postoperative day 20, repeat blood culture was negative, whereas necrotic tissue culture remained positive for K. pneumoniae. After infectious-disease consultation, antimicrobial therapy was changed to ceftazidime 1 g every 8 h. During the subsequent two operations, tissue samples were collected for bacterial culture and antimicrobial susceptibility testing, and the results consistently showed K. pneumoniae susceptible to ceftazidime. Therefore, this antimicrobial regimen was continued until 5 days after the final operation, followed by an additional 7-day course of oral ceftazidime therapy. Strict glucose monitoring and control, regular wound disinfection, and scheduled dressing care were maintained. After the final procedure, the patient was afebrile and clinically stable. The wounds had largely healed, with only scattered granulation areas in the perineum and scrotum, minimal discharge, and no obvious surrounding erythema or swelling (Figure 3C). He was discharged in good condition. At 3-month follow-up, he reported no specific discomfort, the surgical wounds had healed satisfactorily, and he had continued standardized glycemic management after discharge. The patient stated that he was satisfied with the diagnostic and therapeutic process throughout his illness and with the final surgical outcome.

## 3 Discussion

FG was historically considered idiopathic, but most contemporary cases are associated with identifiable precipitating factors. These can be broadly grouped into three categories. The first is impaired host immunity, most commonly due to diabetes mellitus, HIV infection, alcohol misuse, leukemia, or other malignancies (10). The second is local urogenital or anorectal disease, including renal abscess, urinary calculi, urethral stricture, perianal abscess, thrombosed external hemorrhoids, or strangulated inguinal hernia, each of which can promote local infection and impaired perfusion (11). The third is iatrogenic inoculation after traumatic or inappropriate medical procedures, which is less common but clinically important (12, 13). In the present case, poorly controlled diabetes created a susceptible host state, while intestinal obstruction with suspected intra-abdominal infection likely contributed to nutritional compromise and impaired immunity. The temporal sequence—mechanical obstruction first, followed by perineoscrotal symptoms after the abdominal symptoms had improved—raises the possibility of a gastrointestinal contribution. The isolation of Klebsiella pneumoniae from both blood and wound cultures supports an enteric source. Klebsiella pneumoniae commonly colonizes the human gastrointestinal tract and can cause extraintestinal infection when host defenses are impaired. The patient had also undergone repeated enema therapy during conservative treatment of intestinal obstruction; mucosal injury and disruption of the intestinal barrier may have facilitated bacterial translocation. These factors may have acted together to drive disease onset and progression. However, it should be noted that the above clinical evidence cannot directly confirm that intestinal obstruction and intra-abdominal infection were the source of infection and direct precipitating factors for FG. No abdominal fluid or tissue was cultured at the referring hospital, and no bowel perforation, fistula, abscess, or direct anatomical tract between the abdominal process and the perineal infection was demonstrated. In addition, the isolate was not assessed by string test, capsular serotyping, or virulence-gene testing; a hypermucoviscous or hypervirulent K. pneumoniae phenotype cannot be excluded. These limitations prevent definitive attribution of the invasive infection solely to diabetes, impaired immunity, or gastrointestinal translocation. Although these limitations exist, this case may still provide valuable insights into the potential precipitating factors of FG. When encountering FG patients with a similar history of intestinal disease and recent intra-abdominal infection, clinicians should consider gastrointestinal and intra-abdominal sources as possible underlying precipitating factors. Actively obtaining evidence of the infection source and conducting further microbiological investigations may help clinicians better understand the underlying causes and mechanisms of disease development, thereby enabling more targeted and individualized treatment.

FG is difficult to diagnose early because initial symptoms are often nonspecific, yet progression can be rapid and lethal. Prognosis is closely linked to the timing of diagnosis and definitive treatment (14). Laboratory-based scoring systems are often used to estimate severity and mortality risk. The Fournier's Gangrene Severity Index (FGSI), the earliest such tool, incorporates vital signs and laboratory parameters including leukocyte count, hematocrit, and serum creatinine; a score of 9 or higher has been associated with mortality up to 75% (15). The Uludag Fournier's Gangrene Severity Index (UFGSI) adds patient age and extent of disease to FGSI and uses the same threshold to improve prognostic discrimination (16). The Simplified Fournier's Gangrene Severity Index (SFGSI), comprising creatinine, hematocrit, and potassium, has reported sensitivity of 87% and specificity of 77% for mortality prediction and can support rapid risk stratification when full assessment is impractical (17). In this patient, the FGSI was 5, whereas the UFGSI was 11 because of the extensive anatomical spread. The infection involved the perineoscrotal region, abdominal wall, posterior abdominal musculature, psoas and iliacus muscles, perirenal fascia, and retroperitoneal spaces. Thus, even when FGSI appears modest, wide fascial extension may identify a high-risk phenotype. Therefore, for patients with FG with extensive involvement suggested by physical examination or imaging findings, UFGSI may provide a more accurate prediction of disease severity compared with FGSI. Currently, no scoring model can comprehensively reflect the heterogeneity of disease severity and clinical course among different patients. Individual patient characteristics, pathogen-related factors, and disease progression patterns may all contribute to different clinical outcomes. Future prognostic models may be strengthened by integrating established precipitating factors, culture and susceptibility profiles, and structured radiological assessment of anatomical spread.

Management of FG requires multidisciplinary coordination, including close monitoring, resuscitation and supportive care, intravenous broad-spectrum antimicrobial therapy, metabolic optimization, and urgent surgical debridement. Debridement must define the full anatomical extent of infection and remove nonviable tissue thoroughly, as incomplete source control worsens prognosis (11). The first debridement is generally recommended within 6–12 h after symptom recognition or diagnosis; even hospitals without advanced intensive care or reconstructive capacity should prioritize immediate surgical source control within this narrow window (18). Deep necrotic tissue should be sampled for microbiological culture and susceptibility testing during the initial operation. Many patients require repeated debridement because the infected field is extensive and tissue viability evolves over time. Surgeons must balance adequate excision against unnecessary tissue loss and bleeding risk. Skin-sparing approaches have therefore gained attention: when infection and necrosis are completely removed, well-perfused superficial skin and soft tissue can be preserved through multiple small incisions, subcutaneous tunnel debridement, or staged procedures (19). Repeated operative assessment enables direct evaluation of wound progression, anatomical spread, and newly necrotic tissue. Wound care typically includes wet-to-dry dressing changes two to three times daily, which supports local cleanliness, healing, and repeated clinical reassessment (18). Definitive reconstruction may involve primary closure, delayed closure, local flap reconstruction, or plastic surgical reconstruction depending on defect size, tension, tissue quality, and contamination (20). This patient underwent comprehensive treatment over more than two months, including combined antimicrobial therapy, glycemic control, five surgical procedures, staged wound closure, and local flap reconstruction. It is noteworthy that after the initial emergency debridement, the patient did not undergo subsequent debridement and wound closure within a short interval. Instead, the wounds were managed with an open approach, including frequent disinfection, irrigation, and assessment of wound conditions. The patient remained hemodynamically stable, without clinical signs of rapidly progressive necrotizing infection. As the acute infection was controlled, the patient's glycemic status, anemia, hypoalbuminemia, electrolyte disturbances, and nutritional status were gradually corrected, and the next debridement procedure was performed 20 days after the initial operation. We believe that in patients with extensive FG involvement, when adequate source control has been achieved during the initial surgery, and close monitoring of wound conditions and systemic status can be maintained after surgery without evidence of ongoing infection progression, staged wound revision and delayed closure may allow further assessment of tissue viability, promote the formation of healthy granulation tissue, and maximize preservation of potentially viable tissues while achieving thorough infection control. However, the timing of subsequent operations should still be determined dynamically according to the patient's local wound condition and systemic status. If progressive necrosis or inadequate infection control occurs, repeat surgical exploration should be performed promptly, and necessary debridement should not be delayed solely to improve nutritional or metabolic status.

## 4 Conclusion

FG is an insidious, rapidly progressive, and potentially fatal necrotizing fasciitis encountered in urology and emergency medicine. This case describes FG that developed after intestinal obstruction complicated by suspected intra-abdominal infection, a potential precipitating setting that has been rarely reported. Although there is no direct clinical evidence confirming that FG was directly caused by intestinal obstruction and intra-abdominal infection, this case may help clinicians recognize and manage FG patients with similar clinical courses in a timely manner. The clinical course underscores the need to integrate imaging-defined disease extent with conventional severity scoring, to perform early and repeated debridement when necessary, and to combine surgical source control with antimicrobial therapy, glycemic management, and meticulous wound care. These principles are particularly relevant for patients with extensive fascial spread.

## Data Availability

The original contributions presented in the study are included in the article/Supplementary Material, further inquiries can be directed to the corresponding author.
